# Effect of Blood Flow Restriction Technique on Delayed Onset Muscle Soreness: A Systematic Review

**DOI:** 10.3390/medicina58091154

**Published:** 2022-08-25

**Authors:** Sandra Rodrigues, Pedro Forte, Eva Dewaele, Luís Branquinho, José E. Teixeira, Ricardo Ferraz, Tiago M. Barbosa, António M. Monteiro

**Affiliations:** 1 FP-I3ID, FP-BHS, Escola Superior de Saúde Fernando Pessoa, Rua Delfim Maia, 334, 4200-253 Porto, Portugal; 2 Department of Sports, Higher Institute of Educational Sciences of the Douro, 4560-708 Penafiel, Portugal; 3 Department of Sports Sciences and Physical Education, Instituto Politécnico de Bragança, 5300-253 Bragança, Portugal; 4 Research Center in Sports, Health and Human Development, 5001-801 Vila Real, Portugal; 5 Department of Sports Sciences, University of Beira Interior, 6201-001 Covilhã, Portugal

**Keywords:** tissue flossing, blood flow restriction, delayed onset muscular soreness, DOMS

## Abstract

*Background and Objectives*: The effect of the blood flow restriction technique (BFR) on delayed onset muscular soreness (DOMS) symptoms remains unclear. Since there is no consensus in the literature, the aim of the present study is to systematically identify and appraise the available evidence on the effects of the BFR technique on DOMS, in healthy subjects. *Materials and Methods*: Computerized literature search in the databases Pubmed, Google Scholar, EBSCO, Cochrane and PEDro to identify randomized controlled trials that assessed the effects of blood flow restriction on delayed onset muscular soreness symptoms. *Results*: Eight trials met the eligibility criteria and were included in this review, presenting the results of 118 participants, with a mean methodological rating of 6/10 on the PEDro scale. *Conclusions*: So far, there is not enough evidence to confirm or refute the influence of BFR on DOMS, and more studies with a good methodological basis are needed, in larger samples, to establish protocols and parameters of exercise and intervention. Data analysis suggests a tendency toward the proinflammatory effect of BFR during high restrictive pressures combined with eccentric exercises, while postconditioning BFR seems to have a protective effect on DOMS. Prospero ID record: 345457, title registration: “Effect of Blood Flow Restriction Technique on the Prevention of Delayed Onset Muscle Soreness: A Systematic Review”.

## 1. Introduction

DOMS is defined as a functional muscle injury due to overexertion [1], more specifically, it is a generalized muscle pain following unaccustomed, eccentric deceleration movements. It has been classified as a type 1B sports’ muscle injury, by the Munich consensus statement [2]. It is usually caused by eccentric muscle contractions that require the stretching of muscle fibers or the practice of unusual and/or intensive exercises [3]. Symptoms include acute inflammatory pain, namely at rest, hours after the onset of activity [2]. DOMS results from the expression of a complex pathophysiological mechanism [4] whose exact cause is not well understood but is believed to involve inflammatory reaction or even muscle damage [5].

In 1977, a document from V. W. Abraham [6] evaluated DOMS from three different perspectives, surface electromyography to evaluate muscle spasm, presence of myoglobinuria to evaluate the possibility of muscle cell damage, while the ratio of hydroxyproline/creatinine (OHP/Cr) in 24 h urine collection was used as a marker for connective tissue involvement, and concluded that the observations supported the concept that exercise-induced soreness may be related to disruption of the connective tissue elements in the muscle and/or their attachments. Furthermore, these changes are thought to be due to reversible microtraumas in the muscle at the level of the normal structure of the muscle fiber, with potentially aggravating associated lesions of the sarcolemma, transverse tubules, and sarcoplasmic reticulum [7], which induce a disorganization of the sarcomere [4]. When the cytoskeleton is damaged, it becomes more permeable, inducing excessive depletion of muscle enzymes such as creatine kinase (CK), lactate dehydrogenase (LDH), or products resulting from connective tissue degradation (i.e., hydroxyproline or hydroxylysine). This emptying will activate a calcium-dependent proteolytic enzyme, which will directly interfere with the production of adenosine triphosphate (ATP) [5,7]. In addition, at the same time, in response to microlesions and with the aim of removing structural damage induced by exercise, an inflammatory process will begin with the combined action of macrophages, neutrophils, bradykinin, high levels of extracellular potassium, prostaglandin, and edema [4].

According to Armstrong, Warren and Warren [8], there are three phases in this process that are the autogenic phase (which occurs three hours after exercise and corresponds to the beginning of the degradation of the injured structures), the phagocytic phase (progression of pressure and internal temperature of the muscle, increased spontaneous discharge of nociceptors and release of the P substance, favoring amplification and self-maintenance of inflammatory response and global hyperalgesia), and finally, the regeneration phase between the 4th and 6th days. The development of clinical symptoms is delayed (i.e., usually after 24 h, with a peak between 48–72 h post-exercise) as the result of complex sequences of physiological, local, and systemic responses, and can last five to seven days [9], thus increasing the risk of injury in this period [7]. Clinical signs and symptoms are mainly pain on palpation and movement, decreased muscle strength and performance, movement restriction, stiffness, edema, and biomechanical alteration of adjacent joints [2,3].

There are several interventions aimed at preventing or relieving symptoms, namely the massage technique [10], compression techniques [11], cryotherapy [12], or contrast baths [13], which have numerous benefits at the level of DOMS. The absence of a known "gold-standard” method and the diversity of treatment techniques available are largely due to the lack of understanding of the exact mechanisms of DOMS [3].

The use of tissue flossing (TF) is a relatively recent treatment modality that gained popularity through the book by Starrett and Cordoza [14]. Indeed, the introduction of floss band (FB) compression to increase the range of motion indicates that the potential mechanisms behind the benefit of TF can be attributed to fascial shear and blood reperfusion to the muscle [15]. The tissue flossing technique is also called Blood Flow Restriction (BFR) or Kaatsu. The mechanisms involved in TF are similar to ischemic preconditioning or BFR training [14,15] in which an application of an external pressure is used, above or below the muscle or appendicular joint, with the application of a tourniquet/inflatable cuff in the most proximal portion of the limb (in the case of BFR) or an elastic band (i.e., in the case of TF) [16,17]. The pressure provided by the technique safely maintains the influx of arterial blood but reduces or occludes the venous flow distal to the site [18]. This will be associated with a subsequent increase in metabolic accumulation in growth hormone release responses, increased muscle strength, and contractability [14,15]. It is therefore hypothesized that the technique will have several benefits, namely at the level of range of motion, improvement in performance [15], reduction of pain and DOMS, prevention of injuries, improvement of muscle recovery [19] or even increase muscle gains [20].

Several systematic reviews were found on the application of the technique and its effects on specific pathologies and musculoskeletal disorders such as osteoarthrosis [21]. After immobilization [22] or after reconstructive surgery of the anterior cruciate ligament [21] for example, but also its effects on aerobic capacity [23], performance, hypertrophy, and increased muscle strength [24], or even in DOMS [25,26] but to date, the evidence of this technique either in favor of inducing DOMS or preventing it, has not been established.

Thus, the aim of this study is to systematically identify and appraise the available evidence on the effects of the BFR technique on DOMS, in healthy subjects.

## 2. Materials and Methods

The research was conducted using the PICO strategy [27] with a defined population: adults, healthy, without disease or musculoskeletal injuries, an intervention with BFR or TF techniques combined with exercise, a comparison including the same exercises but without BFR or TF, and typical outcomes of DOMS. This review followed the recommendations of PRISMA (Preferred Reporting Items for Systematic Reviews) [28].

In the present review, a computerized literature search was performed by two independent researchers, with the following primary keywords: “tissue flossing”, “restricted blood flow”, “delayed onset muscle soreness”, “DOMS” using logical operators (AND and OR) making the following combination (“tissue flossing” OR “blood flow restriction” OR kaatsu) AND (“DOMS” OR “delayed onset muscle soreness”), from inception till December 2021. The included databases were Pubmed, Google Scholar, EBSCO, Cochrane and PEDro. The final selection of articles met the following eligibility criteria: (1) Randomized controlled trials; (2) With no language restriction; (3) Studies evaluating the effects of the BFR technique with cuff occlusion or floss band on the prevention of DOMS; (4) Studies conducted in trained or untrained adult individuals who did not present pathologies or musculoskeletal injuries; (5) Studies whose experimental group has the BFR technique and whose control group does the same training without BFR application; (6) Studies whose application of BFR technique is carried out during or after the end of training; (7) Intervention with a training protocol of endurance, strength or aerobic training; (8) Studies evaluating DOMS at the beginning and several days after exercise, including at least one of these result measures: pain scales, namely the visual analog pain scale (VAS), DOMS scales such as the Likert Scale of Muscle Soreness, measurements of algometry (i.e., pressure pain threshold), measurements of the activity of blood marshes such as CK or LDH, evaluation of muscle strength and function, such as the maximum voluntary isometric contraction, evaluation of edema (e.g., limb girth measurement) and range of motion evaluation. Articles were included from inception to date. To determine the eligibility or exclusion of each study, the titles, and abstracts of all articles and, in case of doubt, the full text was red.

The methodological quality was evaluated by two independent researchers, using the Physical Evidence Database scoring scale (PEDro) whose application allows quick and effective identification of studies that may have internal validity (criteria 2–9) and sufficient statistical information to perform an interpretation of its results (criteria 10–11). The final score is attributed by the sum of the number of criteria classified as satisfactory between 2 and 11, and criterion 1, relative to external validity, is not considered in the calculation. The score can vary between 0 and 10 points and will allow us to have a careful evaluation of randomized controlled studies to include in the performance of systematic reviews [29].

## 3. Results

**Figure 1:**
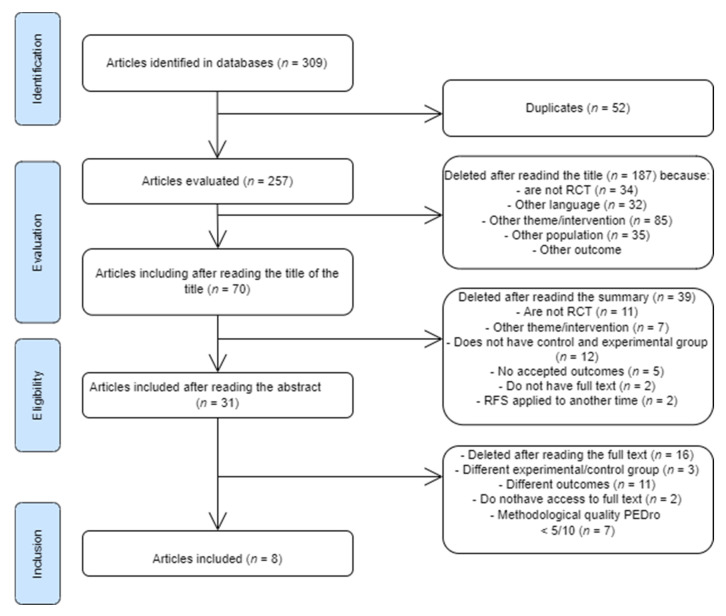
Prisma flowchart of included studies.

During the research carried out in the different databases, a total of 309 articles were found, and this total was reduced to 70 after reading of the titles, and then to 31 post-reading of the abstract. Of these 31 articles, after reading the full text, 8 articles were selected (Figure 1).

### Evaluation of Methodological Quality

The studies present methodological quality with an arithmetic mean of 5.88 out of 10 on the PEDro scale, the result of the evaluation of two independent raters (Table 1).

The summary of the content of the articles is presented in Table 2. The total number of participants was 118, of whom 10 were female and 108 males (the minimum reported sample was 9 elements and the maximum 21) with an arithmetic mean of 15 elements per study and aged between 18 and 39 years.

Brandner e Warmington [30], aimed to determine the perceptual responses to resistance exercise with heavy loads (80% of 1 repetition maximum [1RM]), light loads (20% 1RM), or light loads in combination with BFR. Seventeen healthy untrained males participated in this randomized cross-over study. After four sets of an elbow-flexion exercise, participants reported ratings of perceived exertion (RPE), with DOMS also recorded for seven days after each trial.

Curty et al. [31], aimed to evaluate the acute effects of high-intensity eccentric exercise combined with BFR on muscle damage markers, perceptual and cardiovascular responses. Nine healthy men underwent unilateral elbow extension in two conditions: without and with BFR. The protocol corresponded to three sets of 10 repetitions with 130% of maximal strength (1RM).

Freitas et al. [32], investigated if resistance exercise performed at differing Arterial Occlusion Pressures causes oxidative stress and muscle damage. Twelve males completed 4 sets of 10 repetitions of knee extension at 20% of 1RM, with 30 s rest intervals between sets, that varied only in the amount of restriction pressure applied.

Page, Swan e Patterson [33], examined the effectiveness of intermittent lower limb occlusion in augmenting recovery from exercise-induced muscle damage in physically active males. The sample consisted of sixteen healthy recreationally active male participants who were randomly assigned to an intermittent occlusion (*n* = 8) or control (sham; *n* = 8) group and the protocol consisted of 100 drop-jumps.

Penailillo et al. [34], compared the effects of eccentric cycling and eccentric cycling with blood flow restriction on the changes in cardio-metabolic demand and indirect markers of muscle damage in 21 healthy men, that were randomly allocated into two groups.

Prill, Schulz and Michel [19], investigated if BFR applied to the upper limb, after exercise, would reduce the perception of DOMS, for this, 17 university students underwent an upper limb exertion program and had one of their upper limbs treated afterward.

Thiebaud et al. [35], studied the amount of muscle damage after low-intensity eccentric contractions with blood flow restriction. For this, the authors have compared low-intensity eccentric contractions of the elbow flexors with and without BFR for changes in indirect markers of muscle damage. Nine untrained young men performed the exercises with one arm assigned to BFR and the other without BFR.

Wernbom et al. [36], aimed to investigate muscle activity and endurance during fatiguing low-intensity dynamic knee extension exercises with and without blood flow restriction. For this, eleven healthy subjects with strength training experience performed three sets of unilateral knee extensions till concentric torque failure at 30% of the one repetition maximum. According to the reported protocol, one leg was randomized to exercise with cuff occlusion and the other leg to exercise without occlusion.

## 4. Discussion

The studies included in the present review seem to be clustered into three main categories, those in favor of a pro-inflammatory effect and theoretically greater muscular adaptations to exercise, those in favor of an anti-inflammatory effect, and the ones that advocate no effect of the technique.

Studies in favor of a pro-inflammatory response of the BFR on DOMS. Brandner and Warmington [30] and Penailillo et al. [34], showed a significant increase in different parameters of DOMS in their experimental groups with BFR application compared to the control group. Furthermore, the cross-over study by Brandner and Warmington [30] suggests that the resistance exercise with BFR in elbow flexors, either with a heavy load (80% of 1RM) or with a light load (20% of 1RM), results in greater indicis of DOMS than the exercise without BFR. It was also observed that induced DOMS is higher when BFR is applied with high intermittent pressure than with low continuous pressure, suggesting that high restrictive pressures on muscle tissue can promote the appearance of DOMS. The study by Penailillo et al. [34], using the eccentric cycle ergometer at 60 rpm, combined or not with BFR, did not show significant differences in the level of the pressure pain threshold between the two groups, but there was a later recovery in the initial pain threshold on the EG than in the CG, demonstrating a tendency to induce higher DOMS in exercise under BFR. Moreover, they also observed a slower recovery to the initial levels of MVC and the initial level of muscle pain in the exercise group combined with BFR, when compared to the condition without BFR [30,34].

In these studies that support the pro-inflammatory effect of the BFR technique, the applied pressure was always adapted to each of the participants, thus allowing the achievement of higher levels of accuracy both in the protocol and in the results of the studies [30,34]. In the study by Brandner and Warmington [30], the pressure was defined from 80 to 130% of PSS (i.e., a pressure of 93 ± 2 mmHg at 152 ± 3 mmHg) and Penailillo et al. [34] defined a pressure of approximately 60% of the arterial occlusion (i.e., on average 192 ± 24 mmHg), estimated from the thigh girth of each participant. Despite having different protocols, types of exercises, and BFR pressures, the results of these studies show that eccentric exercise with BFR induces additional effects of mechanical and metabolic stress that induce higher levels of inflammation and thus increase the production of reactive oxygen species during exercise [37], with induction of higher Levels of DOMS and recovery times.

In addition, despite the BFR technique promoting more DOMS, it has been shown that its additional effects of mechanical and metabolic stress may have advantages, since they constitute the first responsible factors for muscle hypertrophy, by demonstrating that the BFR technique promotes strength and muscle mass gain [38].

Studies in favor of an anti-inflammatory effect of the BFR on DOMS. Concurrently, Wernbom [36], Page, Swan and Patterson [33], and Prill, Schulz and Michel [19] found significant results among the experimental and control groups showing a decrease in DOMS due to the application of the BFR technique, namely at the level of the evaluated parameters like pain (in the three studies) and muscle strength and CK levels [33]. The decrease in DOMS suggests a reduction in the inflammatory response (by decreasing the influx of inflammatory mediators) allowed by the BFR technique, thus leading to the reduction of muscle edema and intramuscular pressure, which decreases the sensitivity and stimulation of nociceptors, potentially reducing the sensations of pain, stiffness, and myalgias [39]. Despite having similar conclusions, these three studies show some differences, particularly in the time of technical performance. Prill, Schulz and Michel [19], and Page, Swan and Patterson [33] induced BFR after exercise, called the ischemic postconditioning process, and not during exercise as in the Wernbom study [36]. There are also differences in the exercise protocols: 100 drop-jumps [33], different exercises for biceps to failure [19], or unilateral knee extensions to 30% of 1RM until failure [36]. All studies with anti-inflammatory results used different BFR pressures: 100 mmHg [19,36], elastic band elongation of 50 to 75% of maximum elongation [17], and 220 mmHg [33] that are already predefined, i.e., they are not chosen specifically for the participant.

Besides, Curty et al. [31], also showed that the BFR technique combined with exercise can have preventive effects on DOMS with faster recovery with BFR than without. In this study, the participants had a limb belonging to the experimental group and the contralateral limb belonging to the control group (30 min between the two groups), the exercise consisted of 3 sets of 10 unilateral eccentric extensions of the elbow, at 130% of 1 RM, with 1 min rest between trials. The BFR was used at 80% of arterial occlusion. There were no significant differences between the two groups both in terms of edema and range of motion. However, ROM in the experimental group returned to the initial degree earlier (after 24 h) than in the control group (after 48 h). A significant difference was also found in the experimental group at the level of pain (evaluated by NPS) because at 48 h it presented less DOMS than in the exercise group.

Studies that do not support either the anti-inflammatory effect or the pro-inflammatory effect of the BFR technique [32,35]. In the study by Freitas et al. [32], participants performed four equal training protocols (4 series of 10 unilateral knee extension repetitions with 30 s of rest) each week with different BFR pressures but specific for each participant: without BFR, 50% of the total AOP (66.58 ± 9.72 mmHg), 75% (99.25 ± 14.95 mmHg) and 100% (129.50 ± 18.73). In this study, the maximum isometric contraction was measured at 1 h, 24 h, and 48 h post-exercise and there was no decrease in isometric strength at any time, regardless of the applied pressure. In addition, there was no increase in DOMS either, for any of the exercise conditions in this study. CK and LDH levels were also measured as indirect biomarkers of muscle damage. Clarkson and Hubal [40] stated that CK levels should increase more than 100% after resistance exercise compared to their baseline levels and that they remain elevated for several days after resistance exercise. However, no significant increases in baseline values in CK and LDH levels were observed over time for any of the conditions tested. Although LDH levels have increased significantly for all conditions 48 h post-exercise compared to 24 h post-exercise, this difference is probably due to normal daily variations in blood LDH levels after performing an exercise. This fact is corroborated by the absence of significant difference between the conditions tested at the various moments after the exercise. In addition, it is important to highlight that the authors outlined the study so that the experimental group completed the repetitions until muscle failure while the control group only completed the same number of repetitions as the experimental group without reaching muscle failure since they were not subjected to BFR. These facts could be pointed out as potential study bias.

In addition, Thiebaud et al. [35], performed a study in which the participants had one control upper limb (without BFR) and another experimental limb (with BFR) (30 min of intervals between the two groups) and performed an eccentric contraction protocol of the biceps at 30% of 1RM, 4 series of 30/15/15/15 repetitions with 30 s of rest. The BFR technique was performed throughout the exercise, with a predefined pressure of 35 mmHg initially, until gradually reaching a final pressure of 120 mmHg. In this study, no significant difference was found in the different parameters evaluated, only a decrease in strength of 7% was shown after exercise. Thus, despite having very different exercise protocols and BFR, no significant differences were found between the experimental and control groups in any of the evaluated parameters: i.e., pain, CK and/or LDH levels, muscle strength, ROM, and edema. Thus, both concluded that the exercise combined with BFR neither promotes nor decreases muscle damage and consequently DOMS.

Loenneke, Thiebaud and Abe [41], who examined the mechanisms of muscle damage resulting from BFR and critically evaluated the available literature on the application of BFR, do not support the hypothesis that the application of BFR in combination with low-intensity exercises increased the incidence of muscle damage. Instead, current literature suggests that minimal or no muscle damage occurs with this type of exercise. No prolonged decrease in muscle function, prolonged muscle edema, or dissimilar muscle pain classifications to a low load submaximal control and no elevation in blood biomarkers of muscle damage were observed.

The available evidence makes it impossible to conclude in favor of an anti- or pro-inflammatory response of the BFR technique in the presence of DOMS. As has already been explained, DOMS can occur after exhaustive and/or unusual exercises, particularly in activities involving eccentric muscle contractions that result in pain, inflammation, and edema and it is a complex area of study since there are several factors such as gender, age, nutrition, level of physical conditioning, genetics, and familiarity with the exercise task, which influence the magnitude of the decrease in performance and recovery time after exercise [39]. In addition, a variety of external factors, such as the type of contraction, duration, and intensity of exercise, may also influence the magnitude of the inflammatory response, and the release of muscle proteins into the circulation after muscle damage, caused by exercise [42]. Furthermore, DOMS can be evaluated indirectly using various methods, including blood markers [43], pain scales [44], ROM measurements, or muscle function and strength (Maximum voluntary contraction—MVC) [40]. Possibly the absence of definite conclusions may be due to the aforementioned causes of heterogeneity between the published studies. Nevertheless, the data in the present study seems to indicate that low load exercise (20 to 30% of 1RM) combined with BFR was not a sufficient stimulus to study the benefits of the occlusion technique, and these studies failed to show any difference between the control and experimental groups. On the other hand, high restrictive pressure during eccentric exercise seems to promote greater DOMS and recovery time, while postconditioning BFR seems to have a protective role in DOMS symptoms.

In general, the studies included in this review presented a reduced sample size, consisting mainly of men, with distinctive protocols, both for training and BFR. They had moderate classification in the PEDro scale, and the absence of blindness in the evaluators and participants was frequent. The main limitation of this systematic review was the absence of gray literature, however, the inclusion of google scholar as a database tried to overcome this limitation. Moreover, the choice of keywords could have been another limitation. Nevertheless, the inclusion of numerous synonyms was the strategy to overcome this limitation.

## 5. Conclusions

The results from the present study seem to advocate the choice of high restrictive pressures, specifically designed for each participant, combined with eccentric exercise to induce DOMS and greater recovery time. This pro-inflammatory effect could be used to induce greater adaptations in terms of muscle hypertrophy and strength. Conversely, postconditioning application, with predetermined restrictive pressure, could be linked to a more protective effect on DOMS. This post-exercise application of BFR ranged between three and five minutes protocols and one to three times of occlusion.

However, the results from this literature review suggest that the effect of BFR on DOMS is not consensual and is still a controversial topic in the scientific literature since some studies support the pro-inflammatory effects of the technique, while other studies support the anti-inflammatory effect or no effect. These differences may be due to the dissimilarities between exercise and intervention protocols. In this sense, further studies of good methodological basis are still needed, in larger samples, to establish protocols and parameters of exercise and intervention, as well as to confirm the efficacy of BFR on DOMS.

## Figures and Tables

**Table 1:** Methodological quality of the studies included in the review, according to the PEDro scale.

Author(s)	Present Criteria	PEDro Score
Brandner e Warmington [30]	2, 4, 9, 10, 11	5/10
Curty et al. [31]	2, 4, 9, 10, 11	5/10
Freitas et al. [32]	2, 4, 8, 9, 10, 11	6/10
Page, Swan e Patterson [33]	2, 5, 7, 8, 9, 10, 11	7/10
Penailillo et al. [34]	2, 4, 8, 9, 10, 11	6/10
Prill, Schulz and Michel [19]	1, 2, 3, 5, 7, 8, 10, 11	7/10
Thiebaud et al. [35]	2, 4, 9, 10, 11	5/10
Wernbom et al. [36]	2, 4, 8, 9, 10, 11	6/10

**Table 2:** Sample description table, objectives, intervention, outcomes, results, and conclusion of the 8 studies included in the systematic review.

Author	Sample	Objective	Description of the Intervention	Outcomes	Results	Main Conclusion
**Brandner e Warmington [30]**	N = 17 M untrained healthy/23 ± 3 years. Each performs the 4 different protocols **G1**: HL (80% 1 RM) without BFR **G2**: LL (20% 1 RM) without BFR **G3**: BFR-C: LL with BFR (20% 1 RM) **G4**: BFR-I: LL with BFR (20% 1 RM)	Determine and compare the perception and DOMS responses to resistance training with HL and LL with and without BFR	Exercise protocol *Biceps curl* (2 s of concentric contraction and 2 s eccentric) **G1**: 4 × 6–8 repetitions, 2.5 min rest **G2–4**: 1 × 30 reps and 3 × 15 reps with 30 s rest. BFR protocol: applied to the most proximal part of the arm. Pressure cycle: 50 mmHg for 30 s and then released for 10 s adding 20 mmHg to each inflation until it reaches 80% of resting PSS (**G3**) and 130%/0% at rest time (**G4**).	- **Pain** (NPS) after palpation and movement Baseline Measurements, 24, 48, 72, 96 and 120 h post-exercise	**Pain** - ↑ pain (*p* ≤ 0.05) in **G3** (24 and 48 h post (*p* ≤ 0.01)) and **in G4** (24, 48 and 72 h post (*p* ≤ 0.01)) compared with baseline. - Post-exercise pain = Baseline pain in groups **G1** and **G2** - ↑ pain **G3** and **G4** > **G1** *(p* ≤ 0.01) and **G3** and **G4** > **G2** (*p ≤* 0.05) 24 and 48 h post-exercise - ↑ pain **G4** (*p* ≤ 0.01) > **G1** and **G2** (*p* ≤ 0.05) at 72 h post-exercise.	**The BFR combined with exercise causes higher DOMS. BFR-I causes more DOMS with longer recovery time than BFR-C**
**Curty et al. [31]**	N = 9 M healthy active (26 ± 1 years) **CG** (without BFR) on a member **EG** (with BFR) on the other	Evaluate the acute effect of eccentric exercise with BFR on DOMS markers	Exercise protocol: Unilateral elbow extension (eccentric phase only), 3 × 10 reps at 130% of 1RM, 1 min rest. 30 min between the two groups. BFR protocol: pressure of ≈80% to have complete BFR in resting condition. The pressure was about 121 ± 7 mmHg in the dominant arm and 122 ± 4 mmHg in the non-dominant arm.	- **ROM** - **CIR** - **Pain** (NPS) Measured before, right after, and 24 and 48 h post-exercise.	**CIR** - NS difference between groups ↑ only of CG compared to baseline immediately after exercise (*p* < 0.05) **Pain** - NS difference between groups. However, DOMS is observed in the EG at 48 h after exercise compared to immediately after exercise *(p* < 0.05) **ROM** - It is observed that the EG returns to ROM baseline earlier (Post 24 h) than the CG (after 48 h) *(p* < 0.05)	**There was no significant difference between the groups, however, it is noted that rom recovery occurs earlier in EG than in the CG. Thus, the BFR technique could be of benefit in the prevention of DOMS**.
**Freitas et al. [32]**	N = 20 M healthy and trained/20.58 ± 2.39 years. Each performs the 4 protocols - **G1**: Exercise without BFR (control) - **G2**: Exercise with 50% BFR - **G3**: Exercise with 75% BFR - **G4**: Exercise with 100% BFR	Investigate whether exercise combined with BFR with different pressures causes oxidative stress and muscle damage	Exercise Protocol (**G1–4**) Unilateral knee extension at 20% of 1 RM, 4 × 10 reps (1.5 s each concentric and eccentric phase) 30 s rest BFR Protocol (**G2–4**): *cuff positioned* in the inguinal part of the limb and inflated before the beginning of the first series until the end of the 4th series. The pressure according to % of the total AOP.	- Pain (NPS) - **MVC** (dynamometer) - **CK and LDH levels** (samples) Evaluated at rest and 1, 24 and 48 h after exercise	In all groups, there was an *increase* (*p* = 0.08) in the 24 h MVC after exercise compared to 1 h post-exercise, as well as a lower LDH *level* (*p* < 0.01) 24 h post-exercise than 48 h post-exercise. However, there is no significant difference between the groups at the level of pain, MVC and in CK and LDH levels at 1, 24 or 48 h post-exercise.	**BFR combined with exercise has no effect on DOMS**.
**Page, Swan e Patterson [33]**	N = 16 M healthy and physically active/22.6 ± 2.8 years EG with BFR after exercise (220 mmHg) N = 8 **CG** with BFR after exercise (20 mmHg) N = 8	Evaluate the efficacy of BFR in recovery from exercise-induced muscle damage	Exercise protocol: 100 *drop-jumps* from a 0.6 m box 5 × 20 reps, 2 min rest BFR protocol: applied after exercise 3 × 5 min occlusion/5 min reperfusion. bilaterally in the proximal portion of the leg 220 mmHg (**EG**) 20 mmHg (**CG**)	- **MVC** (myometer) - **CK levels** (samples) - **CIR** - **Pain** (NPS) Evaluated before and 24, 48 and 72 h after exercise	The decrease in MVC is significantly higher in CF than EG at 24, 48 and 72 h after exercise *(p* < 0.05), CK levels are lower (*p* < 0.05) in EG at 24 and 48 h after exercise. For pain despite having a score of DOMS at 24 h post exercise for CG and EG (*p* < 0.05), pain is lower in EG at 24, 48 and 72 h after exercise (*p* < 0.05). There was no significant difference in CIR between the groups.	**The BFR technique applied after exercise decreases DOMS**.
**Penailillo et al. [34]**	N = 21 M healthy and active/24.0 ± 3.2 years **CG** without BFR N = 10 **EG** with BFR N = 10	Compare the effects of an eccentric cycling session with and without BFR at the level of changes in cardiometabolic demand and indirect markers of muscle damage	Exercise protocol: Warm up (30–60 rpm to about 50 W) for 5 min on the eccentric ergometer followed by a 30 min workout always at 60 rpm (participants must resist movement to maintain % of Max Power Output. BFR Protocol: Application to the most proximal portion of each thigh with a pressure of ≈60% of arterial occlusion (estimated from the circumference of the thigh). The mean pressure used was 192 ± 24 mmHg.	- **MVC** (force plate) - **CK** (samples) before and after 48 h - **Pain** (NPS) - **PPT** (algometer) - **ROM** (AKE e Naclash test) Measured before, soon after, and 24, 48, 72 and 96 h post-exercise	- MVC reduction (*p* < 0.001) **in CG** (24 h and 48 h post) and **EG** (24 h, 48 h and 72 h post) compared with baseline - ↑ CK at 48 h for **CG** and **EG** (*p* < 0.05) compared with baseline. - ↑ pain in **CG** and **EG** at 24 and 72 h após exercise (*p* < 0.001). ↑ 60 % higher EG pain compared with CG (*p* < 0.01) - PPT immediately after the exercise up to 48h (RF) and up to 96 h (VL and VM) in **EG** and **CG** *(p* < 0.001) - AKE TEST: ROM (*p* < 0.001) between 24 h post exercise in the **CG** (*p* < 0.05) and up to 48h post-exercise in the **EG** (*p* < 0.05) *Naclash test*: ROM (*p <* 0.001) in the **CG** and **EG**) between 24 and 48 h post exercise (*p* < 0.05)	**There was a reduction in MVC, PPT and ROM and an increase in CK and pain in both groups, however there is a greater increase in pain in the EG than in the CG and a longer ROM recovery time in the EG than the CG. Thus, the BFR technique combined with exercise induces greater DOMS**.
**Prill, Schulz and Michel [19]**	Healthy, trained N = 15 (7 F and 8 M)/21.9 years (±2.3) 1st day Arm D/ND receives BFR, and another arm serves as CG. 2nd day arm that received BFR 7 days ago is CG, and the other receives the BFR	Assess whether the technical application of TF after exercise can reduce DOMS	Training protocol: Difficult exercises for the biceps 3 × 5–8 repetitions until failure, 1 min rest BFR Protocol: TF, 15 min after training around the arm (at 50 and 75% of maximum elongation) for 3 min combined with elbow (flexion/extension) and shoulder (RI with AB/RE pronation) movements.	- **Pain** (NPS) (after TF, 24 h and 48 h post-exercise)	**62% of the participants had lower DOMS with TF than without, at 24 h (*p* = 0.036) and 48h (*p* = 0.035) after exercise**.	The TF technique plus exercise induces lower DOMS.
**Thiebaud et al. [35]**	N = 9 M active, but untrained/between 18–26 years. BFR group on one arm CG without BFR on the other arm	Evaluate the effects of BFR on indirect DOMS markers	Exercise protocol: Only eccentric contraction (2 s) of elbow flexors at 30% of 1RM, 4 × 30/15/15/15 reps, 30 s rest. 30 min of rest between the two groups. BFR Protocol: With initial pressure of 35 mmHg gradually increased to a final pressure of 120 mmHg	- **MVC** (dynamometer) - **ROM** - **CIR** - **Pain** (NPS) Measured before, just after and 1, 2, 3, and 4 days after exercise	- NS differences found between groups in **MVC**, **ROM**, **CIR** or **pain**. - 7% MVIC immediately after exercise, but then returns to baseline at 24 h after exercise.	**The BFR technique combined with exercise has no effect on DOMS**.
**Wernbom [36]**	N = 11 (8 M and 3 F) trained/between 20–39 years. Each participant has one control leg: training without BFR (C**G**) and another experimental training with BFR (E**G**)	Investigate the differences in activity and muscle hardening in exercise with/without BFR.	Exercise protocol: Unilateral knee extension 30% of 1RM of 3× maximum reps (up to failure), 45 s rest (1.5 s for eccentric and concentric phase). BFR Protocol: applied at a pressure of 100 mmHg before exercise until the end of the	- **Pain** (NPS) - Evaluated 24, 48 and 72 h post-exercise	- Statistically significant difference (*p* < 0.05) at pain level: **EG < CG** at 24, 48 and 72 h after exercise	**Pain is significantly lower in EG than in CG. The BFR combined with exercise relieves DOMS symptoms**.

Subtitles: AKE: Active Knee Extension; ROM: range of motion; AOP: arterial occlusion pressure; BFR-C: blood flow restriction with low continuum pressure; BFR-I: blood flow restriction with high intermittent pressure; CIR: circumference; MVC: Maximum voluntary contraction; D: dominant; ND: non-dominant; NPS: numeric pain scale; G: group; CG: control group; EG: experimental group; M: men; HL: high-load; LL: light-load; W: women; N: sample size; NS: not significant, PPT: pain pressure threshold; SBP: systolic blood pressure; S: significant; RM: repetition maximum; RF: rectus femoris muscle; VL: vastus lateralis; VM: vastus medialis muscle.

## Data Availability

Not applicable.

## Abbreviations

1RM	1 repetition maximum
AKE	Active Knee Extension
AOP	arterial occlusion pressure
ATP	adenosine triphosphate
BFR	blood flow restriction technique
BFR-C	blood flow restriction with low continuum pressure
BFR-I	blood flow restriction with high intermittent pressure
CG	Control group
CIR	Circumference
CK	creatine kinase
D	Dominant
DOMS	delayed onset muscular soreness
EG	Experimental group
G	Group
HL	High load
LDH	lactate dehydrogenase
LL	Light-load
M	Men
MVC	Maximum voluntary contraction
*n*	Sample size
ND	Non-dominant
NPS	Numeric pain scale
ns	Non-significant
PPT	Pressure pain threshold
RF	Rectus femoris
ROM	range of motion
RPE	ratings of perceived exertion
SBP	Systolic blood pressure
S	Significant
TF	tissue flossing
VAS	visual analog pain scale
VL	Vastus lateralis muscle
VM	Vastus medialis muscle
W	Women
